# Supplementary material for: Non-Random Inversion Landscapes in Prokaryotic Genomes Are Shaped by Heterogeneous Selection Pressures
Source: Mol Biol Evol. 2017 Apr 12;34(8):1902–11. doi: 10.1093/molbev/msx127 (PMC5850607; doi:10.1093/molbev/msx127)
Supplement: Supplementary Data [file msx127_Supp.zip › Supplementary_Information.pdf]

Supplementary Information

Supplementary Table 1. Proteobacteria with experimentally determined origin positions.

Taxon (GenBank file name)	GenBank identifier of the largest chromosome	Reference
Azotobacter_vinelandii_DJ_uid16	CP001157.1	[1]
Caulobacter_crescentus_uid298	AE005673.1	[2]
Caulobacter_crescentus_NA1000_uid32027	CP001340.1	[3]
Coxiella_burnetii_uid41	AE016828.2	[4]
Enterobacter_aerogenes_KCTC_2190_uid66537	CP002824.1	[5]
Erwinia_carotovora_atroseptica_SCRI1043_uid350	BX950851.1	[6]
Escherichia_coli_K_12_substr__MG1655_uid225	U00096.3	[7]
Helicobacter_pylori_26695_uid233	AE000511.1	[8]
Klebsiella_pneumoniae_NTUH_K2044_uid21069	AP006725.1	[5]
Neisseria_gonorrhoeae_FA_1090_uid23	AE004969.1	[9]
Pseudomonas_aeruginosa_uid331	AE004091.2	[10]
Pseudomonas_putida_KT2440_uid267	AE015451.1	[10]
Rickettsia_prowazekii_uid43	AJ235269.1	[11]
Salmonella_typhimurium_LT2_uid241	AE006468.1	[12]
Sinorhizobium_meliloti_uid19	AL591688.1	[13]

Vibrio_cholerae_uid36	AE003852.1	[14]
Vibrio_harveyi_ATCC_BAA_1116_uid19857	CP000789.1	[15]
Xanthomonas_campestris_ATCC_33913_uid296	AE008922.1	[16]
Xylella_fastidiosa_uid271	AE003849.1	[17]

Supplementary Table 3. Replisome co-localization propensities across species. Tabulated is the proportion of cases where a single fluorescent focus or two foci were observed in the same cell in a given replisome tracking experiment. Cells with more than 2 foci were assumed to represent re-replication events prior to cell division. These cells and cells without a single focus were excluded and the fraction of cells with 2 foci re-calculated as indicated in column 3.

Species	% 1 focus	% 2 foci	Fraction of cells with 2 foci (f): 2f/(1f+2f)	Reference
E. coli	22	53	0.71	[18]
M. smegmatis	57	27	0.31	[19]
H. pylori	82	9.38	0.1	[20]
C. crescentus	98	2	0.02	[21]
M. xanthus	45	29	0.39	[22]
P. aeruginosa	93	7	0.07	[23]

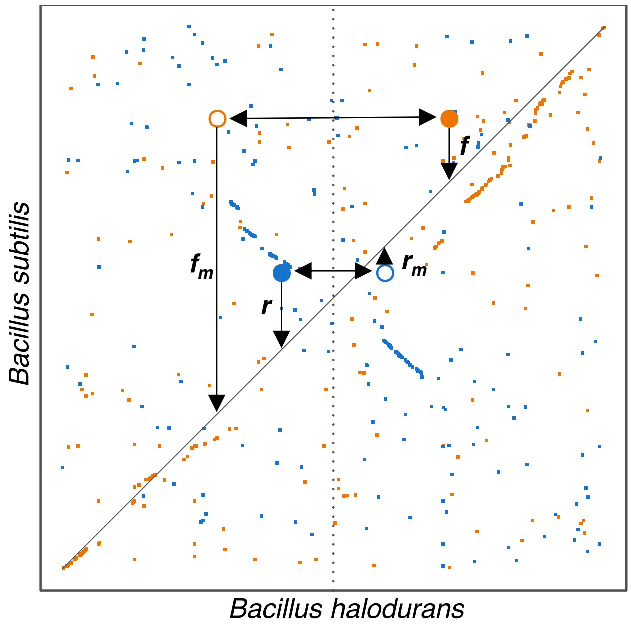

Supplementary Figure 1. Detecting symmetric inversion bias from a MUMmer alignment dot plot. Orange dots represent forward sequence matches (MUMs) between the two genomes, blue dots represent reverse matches. Larger circles give two examples of how each match is treated during calculation of $X_{i,j}$. We measure the absolute residual distance f of the forward match (filled orange circle) from the alignment diagonal and the distance f_m of the mirrored forward match (empty orange circle). The same applies to reverse matches (r and r_m). These distances are then used to calculate the $X_{i,j}$ score according to Eq. 1 in the main text. Importantly, under a symmetric inversion model, we expect r_m to be smaller than r and f to be smaller than f_m. Eq. 1 exploits this: when r_m values are small and f_m values are large, $X_{i,j}$ becomes larger. We compute residuals for all eligible MUMs as described in Materials and Methods, moving the "crosshairs" (alignment diagonal + vertical dotted axis) through the alignment landscape at a resolution of 10 kb, and calculating $X_{i,j}$ at each position.

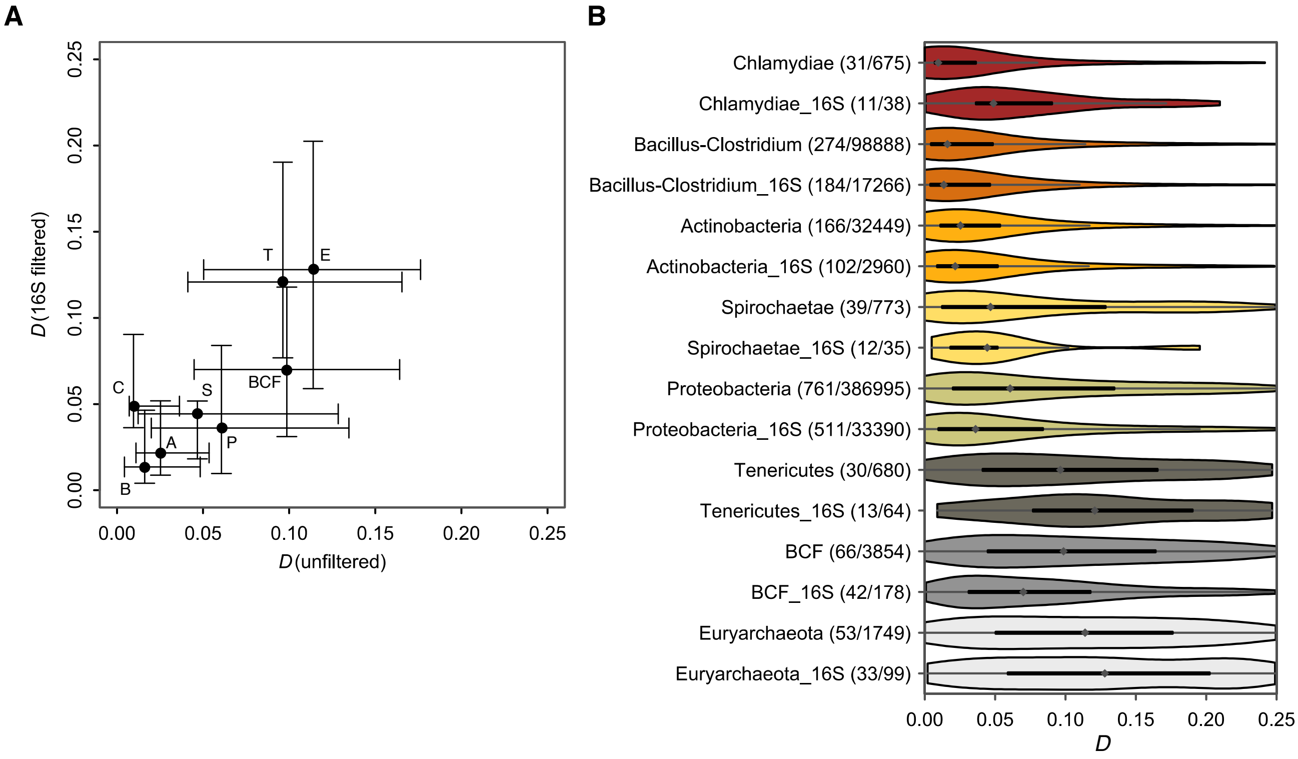

Supplementary Figure 2. **Comparing 16S-filtered and –unfiltered datasets.** (A) Median symmetry scores (*D*) and interquartile ranges for each phylum considering either all pairwise genome comparisons (unfiltered) or a subset of pairwise comparisons sampled to match a common underlying distribution of 16S divergence levels (16S filtered). (B) Filtered and unfiltered distributions of *D* values. Unfiltered distributions correspond to Figure 2 in the main text. C: Chlamydiae, B: Bacillus-Clostridium, A: Actinobacteria, S: Spirochaetae, P: Proteobacteria, T: Tenericutes, E: Euryarchaeota, BCF: Bacteroidetes-Chlorobi-Fibrobacteres.

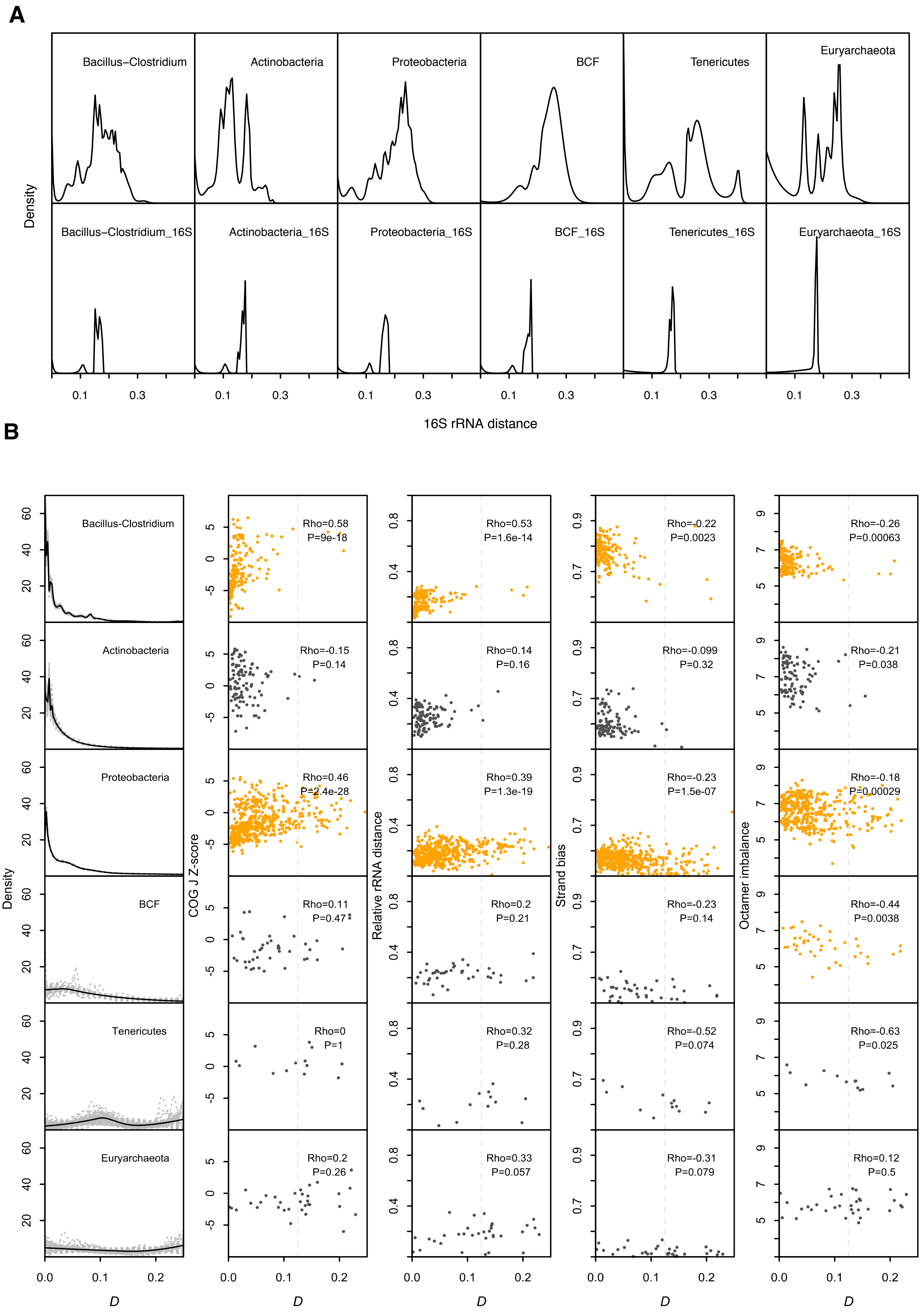

Supplementary Figure 3. Controlling for differential divergence levels across clades. (A) Distribution of 16S rRNA distances for 16S-unfiltered and -filtered (_16S) clades. (B) Relationship between *D* and different features of *ori-ter* related adaptive genome architecture for 16S-filtered datasets. Results for unfiltered data are shown in Figure 4.

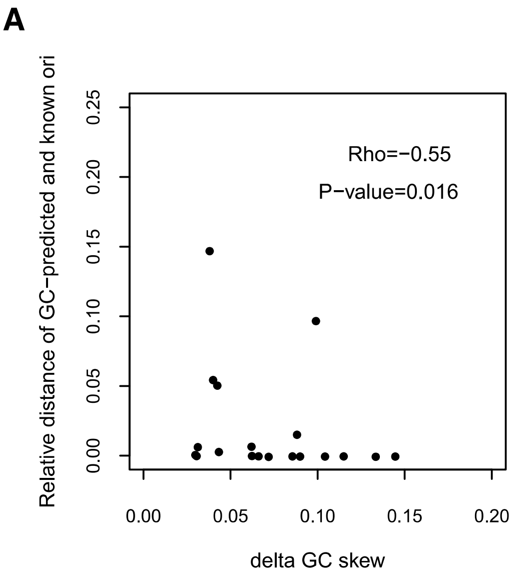
A
Rho=−0.55
P−value=0.016
Relative distance of GC−predicted and known ori
delta GC skew
0.00
0.05
0.10
0.15
0.20
0.25

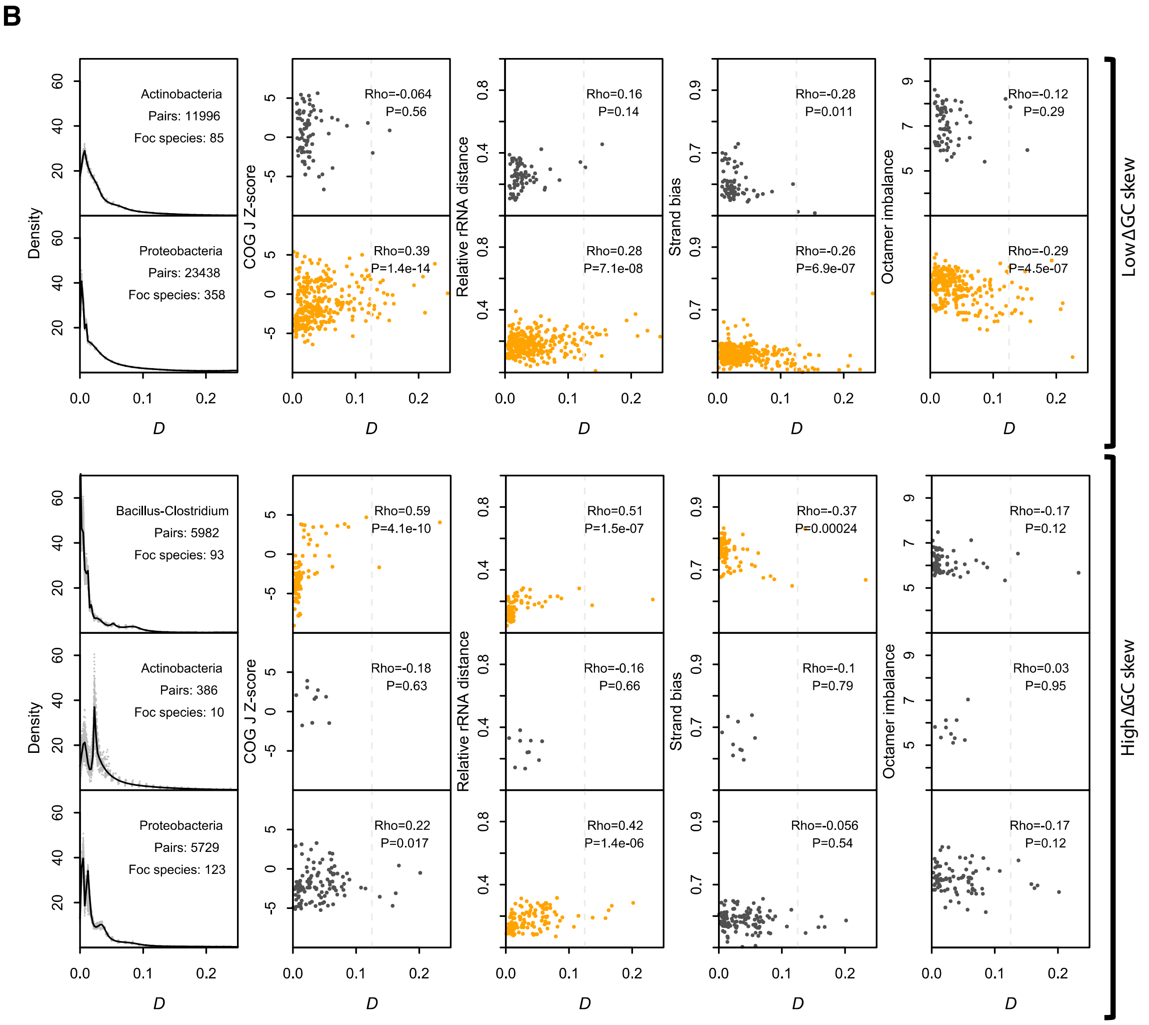
B
Actinobacteria
Pairs: 11996
Foc species: 85
Proteobacteria
Pairs: 23438
Foc species: 358
Density
COG J Z-score
Relative rRNA distance
Strand bias
Octamer imbalance
Rho=-0.064
P=0.56
Rho=0.39
P=1.4e-14
Rho=0.16
P=0.14
Rho=0.28
P=7.1e-08
Rho=-0.28
P=0.011
Rho=-0.26
P=6.9e-07
Rho=-0.12
P=0.29
Rho=-0.29
P=4.5e-07
Low ΔGC skew
D
Bacillus-Clostridium
Pairs: 5982
Foc species: 93
Actinobacteria
Pairs: 386
Foc species: 10
Proteobacteria
Pairs: 5729
Foc species: 123
Rho=0.59
P=4.1e-10
Rho=-0.18
P=0.63
Rho=0.22
P=0.017
Rho=0.51
P=1.5e-07
Rho=-0.16
P=0.66
Rho=0.42
P=1.4e-06
Rho=-0.37
P=0.00024
Rho=-0.1
P=0.79
Rho=-0.056
P=0.54
Rho=-0.17
P=0.12
Rho=0.03
P=0.95
Rho=-0.17
P=0.12
High ΔGC skew

Supplementary Figure 4. Origin mis-predictions do not systematically affect results. (A) ΔGC skew predicts *ori* locations in Proteobacteria with experimentally determined origins. Predictions are better on average when ΔGC skew is large. (B) Relationship between *D* and different features of *ori-ter* related adaptive genome architecture for datasets filtered for ΔGC skew of the focal species and 16S rRNA distances. Low ΔGC skew corresponds to the value range 0-0.1, high ΔGC skew to the range 0.1-0.2. Correlations below a P value threshold of 0.005 are highlighted in orange. See main text for a description of the covariates. Note that ΔGC skew values in the Bacillus-Clostridium clade are almost universally >0.1 so that this clade is not shown in the low ΔGC skew plot.

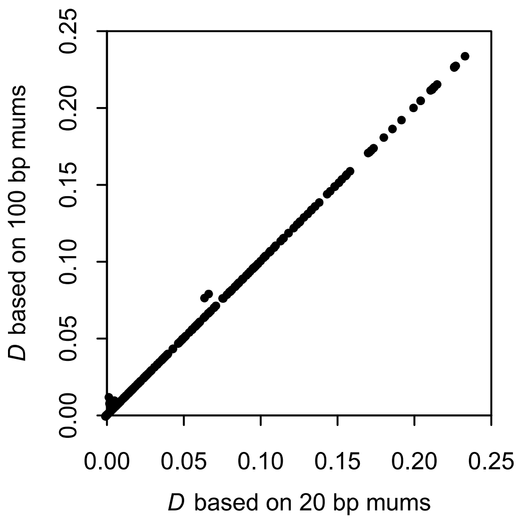

Supplementary Figure 5. Comparison of *D* values calculated based on 20bp and 100bp MUMs for Proteobacteria with experimentally determined origins of replication.

Supplementary References

1. Singh, R. A., Choudhury, N. R. & Das, H. K. The replication origin of *Azotobacter vinelandii*. *Mol. Gen. Genet. MGG* **262,** 1070–1080 (2000).

2. Dingwall, A. & Shapiro, L. Rate, origin, and bidirectionality of *Caulobacter* chromosome replication as determined by pulsed-field gel electrophoresis. *Proc. Natl. Acad. Sci. U. S. A.* **86,** 119–123 (1989).

3. Brassinga, A. K. & Marczynski, G. T. Replication intermediate analysis confirms that chromosomal replication origin initiates from an unusual intergenic region in *Caulobacter crescentus*. *Nucleic Acids Res.* **29,** 4441–4451 (2001).

4. Suhan, M. *et al.* Cloning and characterization of an autonomous replication sequence from *Coxiella burnetii*. *J. Bacteriol.* **176,** 5233–5243 (1994).

5. Harding, N. E. *et al.* Chromosomal replication origins (*oriC*) of *Enterobacter aerogenes* and *Klebsiella pneumoniae* are functional in *Escherichia coli*. *J. Bacteriol.* **152,** 983–993 (1982).

6. Takeda, Y., Harding, N. E., Smith, D. W. & Zyskind, J. W. The chromosomal origin of replication (*oriC*) of *Erwinia carotovora*. *Nucleic Acids Res.* **10,** 2639–2650 (1982).

7. Yasuda, S. & Hirota, Y. Cloning and mapping of the replication origin of *Escherichia coli*. *Proc. Natl. Acad. Sci. U. S. A.* **74,** 5458–5462 (1977).

8. Donczew, R., Weigel, C., Lurz, R., Zakrzewska-Czerwińska, J. & Zawilak-Pawlik, A. *Helicobacter pylori oriC*—the first bipartite origin of chromosome replication in Gram-negative bacteria. *Nucleic Acids Res.* **40,** 9647–9660 (2012).

9. Tobiason, D. M. & Seifert, H. S. The obligate human pathogen, *Neisseria gonorrhoeae*, is polyploid. *PLoS Biol* **4,** e185 (2006).

10. Yee, T. W. & Smith, D. W. *Pseudomonas* chromosomal replication origins: a bacterial class distinct from *Escherichia coli*-type origins. *Proc. Natl. Acad. Sci.* **87,** 1278–1282 (1990).

11. Brassinga, A. K. C. *et al.* Conserved response regulator CtrA and IHF binding sites in the α-Proteobacteria *Caulobacter crescentus* and *Rickettsia prowazekii* chromosomal replication origins. *J. Bacteriol.* **184,** 5789–5799 (2002).

12. Zyskind, J. W. & Smith, D. W. Nucleotide sequence of the *Salmonella typhimurium* origin of DNA replication. *Proc. Natl. Acad. Sci. U. S. A.* **77,** 2460–2464 (1980).

13. Sibley, C. D., MacLellan, S. R. & Finan, T. The *Sinorhizobium meliloti* chromosomal origin of replication. *Microbiology* **152,** 443–455 (2006).

14. Egan, E. S. & Waldor, M. K. Distinct replication requirements for the two *Vibrio cholerae* chromosomes. *Cell* **114,** 521–530 (2003).

15. Zyskind, J. W., Cleary, J. M., Brusilow, W. S., Harding, N. E. & Smith, D. W. Chromosomal replication origin from the marine bacterium *Vibrio harveyi* functions in *Escherichia coli*: *oriC* consensus sequence. *Proc. Natl. Acad. Sci. U. S. A.* **80,** 1164–1168 (1983).

16. Yen, M.-R. *et al.* *oriC* region and replication termination site, *dif*, of the *Xanthomonas campestris* pv. campestris 17 chromosome. *Appl. Environ. Microbiol.* **68,** 2924–2933 (2002).

17. Monteiro, P. B. *et al.* Stable transformation of the *Xylella fastidiosa* citrus variegated chlorosis strain with *oriC* plasmids. *Appl. Environ. Microbiol.* **67,** 2263–2269 (2001).

18. Reyes-Lamothe, R., Possoz, C., Danilova, O. & Sherratt, D. J. Independent positioning and action of *Escherichia coli* replisomes in live cells. *Cell* **133,** 90–102 (2008).

19. Santi, I. & McKinney, J. D. Chromosome organization and replisome dynamics in

Mycobacterium smegmatis. *mBio* **6,** e01999–14 (2015).

20. Sharma, A., Kamran, M., Verma, V., Dasgupta, S. & Dhar, S. K. Intracellular locations of replication proteins and the origin of replication during chromosome duplication in the slowly growing human pathogen *Helicobacter pylori*. *Journal of Bacteriology* **196,** 999–1011 (2014).

21. Jensen, R. B., Wang, S. C. & Shapiro, L. A moving DNA replication factory in *Caulobacter crescentus*. *The EMBO Journal* **20,** 4952–4963 (2001).

22. Harms, A., Treuner-Lange, A., Schumacher, D. & Søgaard-Andersen, L. Tracking of chromosome and replisome dynamics in *Myxococcus xanthus* reveals a novel chromosome arrangement. *PLoS Genet.* **9,** e1003802 (2013).

23. Vallet-Gely, I. & Boccard, F. Chromosomal organization and segregation in *Pseudomonas aeruginosa*. *PLoS Genet.* **9,** e1003492 (2013).
